# Assessing the effect of an educational intervention on early childhood development among Mexican preschool children in the state of Oaxaca: a study protocol of a cluster randomized stepped-wedge trial

**DOI:** 10.1186/s13063-022-06024-2

**Published:** 2022-02-08

**Authors:** Amado D. Quezada-Sánchez, Evelyn Fuentes-Rivera, Angélica García-Martínez, María del Carmen Hernández-Chávez, Carlos Pineda-Antúnez, Martín Romero Martínez, Armando García-Guerra, Raquel García-Feregrino, Abby Madrigal-Ramírez, Tania Santiago-Angelino, Fabián Olvera-Flores, Lourdes Schnaas, Rafael Pérez-Escamilla, Edson Serván-Mori

**Affiliations:** 1 Center for Evaluation and Surveys Research, National Institute of Public Health, Cuernavaca, Mexico; 2 Center for Demographic, Urban and Environmental Studies, College of Mexico A.C., Mexico City, Mexico; 3 Lucy Family Institute for Data and Society, University of Notre Dame, Notre Dame, IN USA; 4 National Institute of Perinatology Isidro Espinosa de los Reyes, Mexico City, Mexico; 5 Center for Health Systems Research, National Institute of Public Health, Universidad Av, 655 Cuernavaca, Mexico; 6 Center for Research in Nutrition and Health, National Institute of Public Health, Cuernavaca, Mexico; 7 Non-Governmental Organization Un Kilo de Ayuda A.C., Mexico City, Mexico; 8 Department of Social & Behavioral Sciences, Yale School of Public Health, Yale University, New Haven, USA

**Keywords:** Stepped-wedge design, Impact evaluation, Early childhood development, NGO, Social vulnerability, Mexico

## Abstract

**Background:**

Early childhood development (ECD) is essential in human capacity building and a critical element in the intergenerational process of human development. In some countries, social programs targeted at improving ECD have proven to be successful. Oaxaca is one of the States with the greatest social inequities in Mexico. Therefore, children in Oaxaca are at a high risk of suboptimal ECD. In 2014, the non-governmental organization (NGO) Un Kilo de Ayuda started to implement the Neurological and Psycho-affective Early Childhood Development Program in eighty marginalized communities of Oaxaca. In this article, we present the impact evaluation design to estimate the effect of this program on ECD.

**Methods:**

We will use a cluster randomized stepped-wedge design with an allocation ratio of 1:1. Communities will be randomly assigned to each study group: four groups of twenty communities each. We expect that children from intervened communities will show better ECD outcomes.

**Discussion:**

This study is one of the few rigorous assessments of the effect of an ECD program on the neurodevelopment of Mexican children recruited in their first 3 years of life from communities of high social vulnerability. Our study design is recommended when the way in which outcomes are measured and assessed depends on age, self-selection is present, and assignment is performed at an aggregate level. Implementation research will be conducted prior to study launch and quality control measures will be in place to maximize the fidelity of study design implementation.

**Trial registration:**

ClinicalTrials.gov NCT04210362

## Administrative information

Note: the numbers in curly brackets in this protocol refer to SPIRIT checklist item numbers. The order of the items has been modified to group similar items (see http://www.equator-network.org/reporting-guidelines/spirit-2013-statement-defining-standard-protocol-items-for-clinical-trials/).

**Table:** 

Title {1}	A stepped-wedge design to assess the effect of an educational intervention on early childhood development among preschool children in the Mexican state of Oaxaca
Trial registration {2a and 2b}.	ClinicalTrials.gov Identifier NCT04210362
Protocol version {3}	Version 2, March 2020
Funding {4}	Un Kilo de Ayuda, A.C.
Author details {5a}	National Institute of Public Health of Mexico National Institute of Perinatology Isidro Espinosa de los Reyes
Name and contact information for the trial sponsor {5b}	Amado D. Quezada-Sánchez National Institute of Public Health of Mexico amado.quezada@insp.mx
Role of sponsor {5c}	Role INSP: Study design, management, analysis, interpretation of data, writing a report and submission of any product for publication. Role funders: Data collection and intervention implementation

## Background {6a}

Early childhood development (ECD) is essential in human capacity building and a critical element in the intergenerational process of human development [1]. ECD is multidimensional and influenced by many factors such as genetics, biological status (health and nutrition), the immediate environment (caregiving components), and community characteristics [2]. Sensitive and responsive nurturing care along with education and good nutritional health can improve ECD; however, the most sensitive window of opportunity to advancing ECD, including its social, emotional, and cognitive aspects, is narrow because the greatest developmental benefits and returns on investment are achieved when nurturing care is offered during gestation and the first 3 years of life [3, 4]. Suboptimal ECD affects not only the child, but also society’s social and economic development [5]. Failure to provide nurturing care in early life to the most vulnerable will lead to high subsequent costs due to excess mortality and morbidity as well as in reduced human capital productivity, perpetuating the vicious cycle that leads to ever increasing social and economic inequities [6].

Studies conducted across different countries have shown that social protection policies and programs have been successful at improving ECD. These interventions include childhood care education, promotion of maternal mental health and wellbeing, and conditional cash transfer programs [7, 8]. In Latin America, “Chile Crece Contigo” is an example of a successful multisectoral evidence-based large-scale program. Funded by the Chilean government and emerging from a national consensus in which the civil society participated, the program offers high-quality ECD information for families and healthcare providers among its various health and education benefits [8]. Another example of a large-scale program is “Cuna Mas” in Peru which consists of home visiting interventions aimed at improving parenting practices; it has showed a positive impact in developmental outcomes [9, 10]. In Colombia, Ecuador, and Mexico, existing cash transfer programs have been used to deliver ECD interventions [11]. Multiple studies across the globe, including Jamaica, Pakistan, and Turkey, have shown that incorporating nurturing care elements in interventions improved child development and later adult outcomes [7].

The most rigorous evaluations of ECD interventions have followed experimental designs which are considered the gold standard to estimate effects. Quasi-experimental designs may be used when randomization is not possible due to self-selection amd ethical or logistical considerations. Studies have found that in hard-to-reach communities with high levels of poverty, children live at risk of nutritional deficiencies and suboptimal levels of neurodevelopment [12]. Often governments face difficulties to reach these populations, many of which are geographically isolated. Therefore, non-governmental organizations (NGOs) are key for complementing and expanding the reach of governmental efforts seeking to improving ECD in the most socially isolated communities.

Located in the South of the country, Oaxaca is one of the States with the greatest social inequities in Mexico. In 2018, 66% of its population lived in poverty, and only 16% had access to health services and 27% had major gaps in the education system [13]. Hence, it is not surprising that Oaxaca has a life expectancy at birth lower than the national average [14] and that a large proportion of children may be at risk of suboptimal ECD. Since 1986, the NGO Un Kilo de Ayuda A. C. (UKA) has been involved in preventing child undernutrition in contexts of high poverty in Mexico. In 2014, UKA started to implement the Neurological and Psycho-affective Early Childhood Development Program (NPECDP-UKA) in eighty socially deprived communities of Oaxaca. This program seeks to improve levels of ECD on children from these communities and is one of the three programs constituting UKA’s Integral Model of Early Child Development. The other two programs focus on improving physical development of children and fostering community development, respectively.

Assessing ECD requires addressing serious methodological challenges given its multidimensional nature. Ethical matters are also important; for example, interventions that include already proved beneficial components must be offered to all groups in a research study limiting the possibility of randomization and inclusion of control groups without any intervention. Interventions designed to improve ECD also face logistical challenges, since they typically include more than one component and numerous instruments to assess all its dimensions [15–18]. Furthermore, they require interdisciplinary teams to deliver the interventions and to conduct unbiased assessments.

This paper aims to present the impact evaluation protocol to assess the effect of the NPECDP-UKA on ECD in preschool children from eighty high social deprived communities in Oaxaca, Mexico. The evaluation has the potential to visualize the effects of an educational intervention performed by an NGO on ECD. It represents an opportunity to assess the developmental lag in the studied communities as well as to provide elements for the continuation, expansion, or modification of the interventions. As part of the civil society and in coordination with authorities, UKA provides a channel to deliver ECD parenting education focused on responsive caregiving. Given the multiple aspects of nurturing care, it is important to have in place multisectoral interventions [19]. Along with the important role from the government, the private sector as well as the civil society can add coordinated contributions to improve and sustain ECD interventions.

The present protocol shows a novel way to assess the effects of an intervention on developmental outcomes where difference scores are not possible due to the age-specific nature of developmental scales. The proposed stepped-wedge experimental design overcome this difficulty and tackle different sources of biases from self-selection, cohort, and community effects. Additionally, the quasi-experimental component of the evaluation allows to study determinants of participation and controls for community and cohort effects. The design could be adapted and applied for studying any other outcome for which age-specific scales are used. Furthermore, it provides useful elements for designing future evaluations by making explicit important biases that may be at play.

To the best of our knowledge, this evaluation study and its design is the first effort of its kind applied to ECD outcomes in Mexico. We hypothesized that children from intervened communities will show better ECD outcomes.

## Methods

### Design and setting {9}

To assess the effect of NPECDP-UKA on ECD, we will use a cluster randomized stepped-wedge design [20] with an allocation ratio of 1:1. A total of 80 communities will be randomly assigned to four study groups using blocking. Each block will be comprised of four communities (twenty blocks in total) with a similar percentage of indigenous population, social marginality level, and urbanicity measured in the 2010 Census [21, 22]. Within each block, the four treatments will be randomly allocated to communities (each community receiving exactly one treatment).

For ethical reasons, interventions will not be allocated at the individual level. Instead, all study groups consist of communities in which eligible caregivers will be invited to enroll in the NPECDP-UKA, but the program will be deployed sequentially at the community level, according to the timing randomly assigned. This defines the distinctive characteristic between study groups, for example, whereas group A will have a total of 30 months of the exposure to the program at its last assessment; group D, the last study group to be incorporated, will have no exposure to the program during the study and will be measured only once (Table 1). In all study groups, a baseline assessment will be performed before implementing the intervention.

**Table 1:**
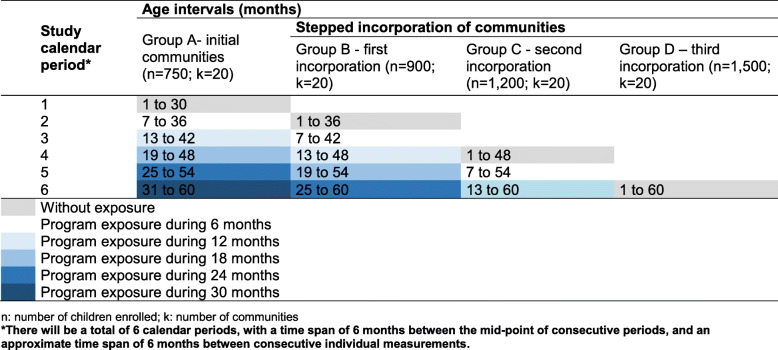
Study groups, exposure times, and age of children

The main feature of the proposed design is the possibility of comparing groups of children at the same age range but with a different time of exposure to NPECDP-UKA, at the same calendar period. This design feature will allow to eliminate potential confounding cohort effects since children with the same age across study groups will also share the same year of birth. Table 1 shows the age range of children for each study group at different study calendar periods. The upper age limit of the ECD assessment will be 60 months; therefore, the first group of communities to be exposed to the program will include children aged 1–30 months at their first assessment and these children will be aged 31–60 at their final assessment. At given calendar periods (*t* = 2, 4, 6), the group unexposed to the intervention will function as a control group. For example, the last group of communities to be incorporated to the study (group D) will function as a control group at the last calendar period (*t* = 6). This will allow to estimate the effect of NPECDP-UKA on ECD for exposure times of 30 months (group A vs group D), 24 months (group B vs group D), and 12 months (group C vs group D). Given the rapid changes at early ages and considering the first thousand days of life as a critical opportunity window, we consider that the planned exposure times are adequate to detect changes as well as a gradient of the effect with respect to time exposure. Comparisons between groups will be performed cross-sectionally for children of the same age range. There will be a total of six calendar periods, with a time span of 6 months between the mid-point of consecutive periods and an approximate time span of 6 months between consecutive individual measurements.

**Fig. 1:**
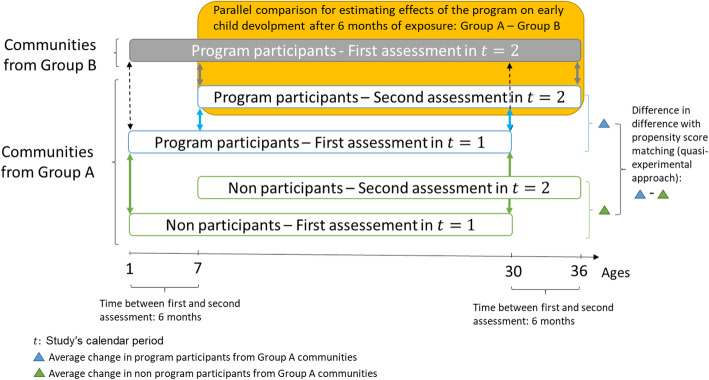
Simplified cluster randomized stepped-wedge and a quasi-experimental design

In addition to the assessment of the effect of NPECDP-UKA on ECD through a cluster randomized stepped-wedge design, children from the initial communities (group A) but whose caregivers refuse to participate in the NPECDP-UKA and continue participating in the study measurements will be assessed at the same time as the intervened children for three consecutive measurements. This will allow to identify predictors of participation and approximate program effects through a quasi-experimental analysis after 6 and 12 months of intervention using propensity score matching techniques to adjust for self-selection predictors [23]. For outcome variables with well-defined changes (e.g., nutritional status indicators), a difference in difference estimator along with propensity score balancing will be used. Figure 1 shows a simplified version including both the cluster randomized stepped-wedge design and the quasi-experimental design. The former is shown just for the comparison between group B and group A in children aged 7 to 36 months at *t* = 2, where group B works as a control group.

### Participants {10}

Communities were selected if they met the following inclusion criteria: located in municipalities where the NPECDP-UKA was not currently operating and with more than thirty-five inhabitants under 5 years, according to the census of 2010 [22]. Within selected communities, children will be included if they match the designed age range, and their caregivers agree to participate in the study. One of the children’s parents or legal caregiver will be asked to sign the study’s consent form by UKA staff (details mentioned below, in the recruitment section). Children’s blood samples will be obtained from capillary blood samples by trained personnel to assess their anemia status. One of the children’s parents or legal caregivers will be asked to sign an additional consent form prior to blood collection. Children whose parents or legal caregivers refuse to provide consent for blood sample collection will not be excluded from the study since the outcomes of interest do not require analysis of blood samples.

### Intervention {11a}

#### Explanation for the choice of comparators

Under the stepped-wedge design framework, communities that will function as a control group will not receive any intervention at the moment of their first measurement. Groups will be compared in a parallel fashion at the same calendar study period, the distinctive feature of study groups is time of exposure to the program, and the group that has not yet been exposed to the program will function as a control group for comparisons at a given study calendar time (see Table 1). Due to ethical considerations, every child, who is diagnosed with undernutrition, anemia, obesity, or developmental delays, will be referred for further assessment and remedial services, regardless of group assignment or program participation status.

#### Intervention details

The NPECDP-UKA is one of the three components constituting UKA’s Integral Model of Early Child Development. The other two components focus on improving physical development of young children and fostering community development, respectively.

#### NPECDP-UKA

UKA will implement an integrated responsive parenting nurturing care approach to promote different child development domains, i.e., motor, language, cognitive, and social. Workshops on appropriate responsive parenting practices will encourage nurturing interactions between parents and/or other caregivers with children, from pregnancy and up to 60 months of age. These workshops are supported by two sets of manuals: the first one is an unpublished pedagogical support guide for the facilitators to deliver program in a standard way, and the set contains the workshop materials needed to support the facilitator’s counseling to families. The model promotes play as a form of learning and addresses responsive parenting skills for healthy feeding, sleeping, soothing, and physical to promote self-regulation of behaviors and emotions.

#### Physical development component

UKA will promote a healthy, balanced, and varied diet and encourage the consumption of locally available foods. Standardized workshops and advice will be implemented to provide guidance on optimal health and nutrition for preschool children (i.e., under 5 years of age). These workshops include a neurodevelopment component and a responsive nurturing care component which covers four dimensions: feeding, sleep, movement, and self-regulation. As part of this component, the implementation team will monitor weight, height, or length, quarterly. Whenever signs of malnutrition are identified, caregivers will be referred to clinical services that may include provision of vitamin supplements and malnutrition recovery advice. Infectious diseases such as diarrhea and acute respiratory infections will also be monitored, and appropriate referrals will be made for clinical services including oral rehydration and counseling on proper hygiene practices. During pregnancy, iron and micronutrient supplementation may be provided if warranted. A comprehensive counseling model will be overseen by community commissioners―contracted by UKA and trained and supervised by the research team―who support the project as translators and interpreters for situations where beneficiaries speak an indigenous language and are not fluent in Spanish. Women will be provided personalized advice during their last trimester of pregnancy and during the first month postpartum. Every 3 months, hemoglobin will be measured in a capillary blood sample for the diagnosis and timely treatment of anemia in pregnant women and children between 6 months and 5 years of age. Treatment of anemia for pregnant women will be iron and folic acid supplementation. Micronutrients will be provided for children at risk of anemia, and prophylactic treatment based on iron will be also provided for children.

#### Community development component

This component has two sub-components. The first addresses household food insecurity. The UKA team will promote access and availability of fresh, healthy, and nutritious food to improve the diet of families with children under 5 years and pregnant women, through local food production based on sustainable community and family farming models. The second sub-component centers around UKA’s effort to provide access to basic WASH services needed for proper ECD including dry ecological toilets, access to water with rainwater collection systems, safe water storage and water purification systems, sludge water treatment systems, and environmentally friendly energy efficient friendly stoves that save wood and decrease the emission of air born pollutants inside the home.

#### Criteria for discontinuing or modifying allocated interventions

The criteria for discontinuing or modifying allocation of communities will be sudden inaccessibility to the community due to external problems such as public insecurity or refusal to participate from local authorities.

#### Strategies to improve adherence to interventions

All training facilitators will participate in training and face-to-face sensitization activities in order to ensure fidelity of program implementation. In order to improve participation rates, before each training program delivery workshop, participants will receive a telephone reminder. During the trial, adherence will be monitored at the beginning and end of each program session.

#### Relevant concomitant care permitted or prohibited during the trial

There are no restrictions on the involvement of participants who receive other interventions such as government programs. We expect that such exposure will be similarly distributed over study groups under random allocation. In case unbalances in this characteristic are detected, participation in other inventions will be adjusted for in analyses.

#### Provisions for post-trial care

The NPECDP-UKA will continue indefinitely after the impact evaluation concludes, but it can be modified as a result of the study to improve its effectiveness.

### Outcomes {12}

#### Primary outcome measures

The primary outcomes of the study are the ECD domains assessed through the Child Development Evaluation Test 2nd Edition (CDE-II). The CDE-II, or *Evaluación del Desarrollo Infantil* (EDI-II) in Spanish, was developed and validated in Mexico to screen populations for risk of developmental delays in early childhood. The test has specific items for fourteen age groups of children aged 1–60 months. ECD domains assessed included gross motor, fine motor, language, social, and cognitive skills. The CDE-II is based on age-group specific items, and score results are categorized into three levels following a traffic light interpretation: green (normal development), yellow (developmental lag), and red (at risk of development delay). These three categories will be used as the specific measurement variable, the analysis metric will be the final value (by ECD domain), and the method of aggregation will be the proportion of children falling within each traffic stoplight category. EDI-II will be carried out according to its application guidelines [24, 25], by trained, standardized, and certified research personnel.

#### Secondary outcome measures

Our secondary outcomes will be child nutritional status and ECD measured assessed through two additional instruments.

#### Nutritional status

Length/height-for-age, weight-for-age, and hemoglobin measurements will be used to assess children’s nutritional status. Anthropometric measurements will be made by trained personnel and standardized according to international protocols [26, 27], using SECA digital scales (874 TM) with an accuracy of ±50 g and SECA portable stadiometers (217 TM) with an accuracy of ±1 mm. After applying data cleaning procedures [28] and following the WHO reference standards [29], our main suboptimal nutritional development indicator will be chronic undernutrition or stunting defined as a length (or height) for age *Z* score below −2.

#### ECD measured through the Bayley Scales of Infant and Toddler Development, Third Edition (BSID-III)

This is a diagnostic test that consists of the following scales: 1) cognitive scale, assesses the child’s non-verbal responses and measures learning processes, problem solving ability, attention, the ability to count and classify objects, and the ability to play 2). Language and communication scale, which includes the subscales to assess receptive and expressive language. The first subscale measures the child’s ability to understand different stimuli, words, or instructions. The second subscale assesses language development through vocalizations, word use, and sentence construction 3). Motor scale, which includes a fine motor subscale that measures hand-eye and hand-to-finger coordination and the gross motor subscale that measures the child’s control over his or her body and abilities to move the torso and limbs 4). Social-emotional scale, which assesses the main milestones of social-emotional development, such as self-regulation, attention, child’s ability to relate to and interact with family members and strangers, among other temperamental and social aspects. These scales are administered and scored independently, resulting in domain-specific assessments. The cognitive, language, and motor scales are assessed through direct observation of the child’s abilities on various items that are ordered in an ascending order of difficulty. The socio-emotional scale comprises thirty-five questions with five Likert-scale-like response points answered by the caregiver.

The BSID-III will be applied in a subsample of children from the group conformed by the first set of communities exposed to the program (group A) and its comparison group of children from caregivers who refused to participate in the study but live in the same communities (i.e., quasi-experimental analyses). Additionally, the BSID-III will be applied in a subsample of group B during its baseline assessment period, i.e., before any NPECDP-UKA exposure occurs. These data will be compared to the subsample of children from group A at their third follow-up, i.e., once they had been exposed for 12 months to the NPECDP-UKA. The BSID-III will be applied to children aged 1 to 42 months.

A concurrent validation using data from children with both BSID-III and CDE-II measures will also be performed.

### ECD measured through McCarthy Scales of Children’s Abilities (MSCA)

An adapted version in Spanish of the original version of the MSCA [30] will be used. This test includes five scales to assess diverse ECD domains: Verbal, Quantitative, Executive-Perceptual, Memory, and Motor. The combination of the first three scales provides a General Cognitive Index (GCI), which is considered equivalent to the IQ. The test will be applied to children from 42 to 60 months of age.

### Participant timeline {13}

Table 2 shows the chronogram of study activities including intervention implementation schedule across study groups and the corresponding measurements of ECD and nutritional status outcomes among pregnant women and children. The number of repeated measurements across time will depend on the timing when each group starts being exposed to the intervention.

**Table 2:** Chronogram of study activities and measurements of child development scales

Activities	Study calendar period						
	t0	t1	t2	t3	t4	t5	t6
Community’s allocation	X						
Eligibility in communities A		X					
Enrollment in communities A		X					
Measurement in communities A*		X	X	X	X	X	X
Eligibility in communities B			X				
Enrollment in communities B			X				
Measurement in communities B*			X	X	X	X	X
Eligibility in communities C					X		
Enrollment in communities C					X		
Measurement in communities C*					X	X	X
Eligibility in communities D							X
Measurement in communities D							X
Measurement of CDE-II		X	X	X	X	X	X
Measurement of MSCA							X
Measurement of BSID-III		X	X	X			
Nutritional status assessment		X	X	X	X	X	X

*Intervention starts right after their first measurement. There will be a time span of 6 months between the mid-point of consecutive periods and an approximate time span of 6 months between consecutive individual measurements

### Sample size {14}

Our main outcome statistic is the proportion of children with developmental lag (yellow category) or at risk of development delay (red category). The effect will be assessed comparing these proportions between the exposed groups and the unexposed group.

Sample sizes were planned so that there are approximately 150 children for each 6-month age interval; this approximates a uniform distribution of observations across age groups and study groups for the relevant age ranges. As mentioned before, given the stepped-wedge design of the study, groups will be incorporated sequentially but comparisons between study groups will be performed at the same study calendar period and for children at the same age interval (see Table 1 for a representation of the stepped-wedge design). Group A will include 1–30-month-old children (*n* = 750); these children will be 31–60 months old at their final measurement and compared to children with the same ages in group D (*n* = 750). This will allow to assess the effect of NPECDP-UKA on ECD after an exposure time of 30 months. Other comparisons are possible. For example, ECD outcomes from group B vs group D (*n* = 900) and group C vs group D (*n* = 1200) at the final study calendar period (Table 1). Table 3 shows the effect size in terms of a difference of proportions given different levels of proportions in the unexposed group (group D), different design effects (DEFF) that consider that measurements in the same community are correlated (DEFF = 1.5, 2.0) [31], a significance level of 0.05 under a bilateral test, and a statistical power of 80.0%.

**Table 3:** Sample sizes and effect sizes in terms of a difference of proportions between two groups given a statistical power of 80%

Sample size per group	Proportion in the group without exposure to NPECDP-UKA	Effect size (difference of proportions) given different design effects (DEFF)*		
		DEFF = 1.0	DEFF = 1.5	DEFF = 2.0
750	0.50	0.072	0.088	0.102
900	0.50	0.066	0.081	0.093
1200	0.50	0.057	0.070	0.081
750	0.25	0.065	0.080	0.093
900	0.25	0.059	0.073	0.085
1200	0.25	0.051	0.063	0.073
750	0.125	0.052	0.064	0.075
900	0.125	0.047	0.058	0.068
1200	0.125	0.040	0.050	0.058

*The design effect is a factor that when multiplied by the variance of an estimator under a simple random sampling design corresponds to its variance under a complex sampling design; in this case, a design with clustering of observations within communities

### Recruitment {15}

There are two important categories for study participants: those who enroll in the NPECDP-UKA and those who refuse to participate in the NPECDP-UKA but still consent in participating with study measurements. Measurements on non-participants will be performed for the first group of communities (group A) to identify predictors of participation and to perform the quasi-experimental evaluation.

Recruitment will be performed in every community in two different stages. The first stage will be based on public convening by the municipal authorities. Local authorities will facilitate the initial contact, aimed at identifying children of interest along with their caregivers, who will be asked about their willingness to participate in the program. The convening will target the population of interest to participate in a meeting where the NPECDP-UKA and its activities will be explained. At these meetings, children and their main caregivers will be identified and their intention to participate in the program will be discussed. For the first group of communities (group A), those who refuse to participate in the program will be asked to participate with study measurements. The second stage of recruitment will be based on the census in the selected municipalities. For this purpose, the housing census provided by the municipal authorities will be used. In homes having children within the designed age range, their primary caregivers will be asked about their intention to participate in the program. Those who refuse to enroll in the program will be asked to participate with study measurements as described before (group A).

The participation of the studied population will be voluntary and written consent will be obtained. The research protocol of this evaluation was approved by the Ethics Committees on Research and Biosafety of the National Institute of Public Health in Mexico (CI-896-2018/1538), and the study is registered in ClinicalTrials.gov (CT/ID: NCT04210362).

### Allocation {16a, 16b, 16c}

The stratification will be made by population size, percentage of the indigenous population, and municipal marginalization. Twenty blocks of four municipalities each will be defined, within which the study group will be assigned through a random-number generator in Stata 15 [32]. The allocation will be carried out at the community level, so it will not be necessary to establish a concealment mechanism. Community allocation will be performed by the National Institute of Public Health Mexico. Within communities, UKA will invite participants to enroll, and they will self-select to 1) enroll in the NPECDP-UKA and the evaluation study, 2) not to enroll in the NPECDP-UKA but participate in the evaluation study, or 3) neither enroll in the NPECDP-UKA nor participate in the evaluation study.

### Blinding {17a, 17b}

The experimental part of our study will be single-blinded. Participants from each community will not know to which group of communities they belong. No procedure for unblinding will be needed. For those collecting and analyzing the data, there will be no blinding given the stepped nature of the study and the defining characteristics of study groups. Regarding the quasi-experimental aspect of the study, there is no blinding since non-participation is the defining characteristic of study groups.

### Data collection and management {18a, 18b, 19}

#### Plans for assessment and collection of outcomes

Data collection of outcomes is planned to occur in the participants’ households by trained personnel not involved in the delivery of the program. There will be up to six assessment timepoints per participant depending on the assigned study group (see the “Participant timeline” section). To promote data quality, besides the training of personnel, there will be duplicate measurements for weight and length (or height).

*Study instruments*

1. The CDE-II, which was developed and validated in Mexico to screen populations for lag and for risk of delay in child development, consists of specific items for fourteen age groups of children aged 1 to 60 months. Assessed developmental areas include gross motor skills, fine motor skills, language, social skills, and cognitive skills. Score results are categorized into three levels: green (normal development), yellow (developmental lag), and red (at risk of development delay) [24].
2. Bayley Scales of Infant and Toddler Development, Third Edition (BSID-III) [33]. This diagnostic test consists of the following scales: (1) cognitive scale, based on the child’s non-verbal responses and measures learning processes, problem solving ability, attention, the ability to count and classify objects, and the ability to play, among other constructs. (2) Language and communication scale, which contains the subscales of receptive and expressive language; the first measures the child’s ability to understand different stimuli, words, or instructions in the environment. The second assesses language development through vocalizations, word use, and sentence construction. (3) Motor scale, which includes the fine motor subscale that measures hand-eye and hand-to-finger coordination and the gross motor subscale that measures the child’s control over his or her body and abilities to move the torso and limbs. (4) Social-emotional scale, which assesses the main milestones of social-emotional development, such as self-regulation, attention, the child’s ability to relate to and interact with family members and strangers, among other temperamental and social aspects. These scales are administered and scored independently, resulting in domain-specific assessments. The cognitive, language, and motor scales are assessed through direct observation of the child’s abilities on various items that are ordered in an ascending order of difficulty. Start (base) and stop (ceiling) criteria determine which test items each child takes. For each item that the child performs correctly, he or she receives a score of 1; if he or she fails to perform the item, the score is 0. The raw score is the sum of correct responses, including items prior to the starting point (base). As mentioned above, the focus of this study is on cognitive, language, and motor development. The socio-emotional scale comprises thirty-five questions of five points each to be answered by the caregiver, so its administration is quick and easy.
3. McCarthy Scales of Children’s Abilities (MSCA). This test is made up of five scales: Verbal, Quantitative, Executive-Perceptual, Memory, and Motor. The combination of the first three scales provides a General Cognitive Index (GCI), which is considered equivalent to the intelligence quotient [34].

We will also collect the following data:

1. Household socioeconomic and demographic characteristics. Includes information on the composition of the household, state of health, education, employment situation, assets, income, social security, and access to social programs of the members living in the same household as the minor of interest.
2. Characteristics of the mother of the selected child. It explores aspects of community organization, participation in organizations, safety in the neighborhood, family support networks, social-emotional characteristics of the mother (depression, stress, anxiety, and self-esteem), opinion on social roles and distribution of tasks within the home, and the mother’s pregnancy history.
3. Characteristics of children from 0 to 30 months. Includes information on pregnancy, delivery and postpartum of the mother of the selected child, addictions of the mother during pregnancy and breastfeeding of the child, health status, nutrition and education of the selected child, and parenting practices (feeding, hygiene, sleep) of the selected child.
4. Knowledge of physical, neurological, and psycho-affective child development. It explores the appropriation of knowledge of the child’s mother from the information presented in the workshops given by the UKA facilitators.
5. Dissemination and acceptance of the UKA program. Collected information on the knowledge, permanence, and desertion of the program by the families of the selected child.
6. Addictions of the members of the household. Explores the risk factors to which the selected child is exposed due to the consumption of licit and illicit substances by members of the household.
7. The last booklet corresponds to Raven’s progressive matrix test [35], applied to the primary caregiver and nuclear family of the selected child.

#### Plans to promote participant retention and complete follow-up

To promote participant retention, a community commissioner will be identified in each community. These commissioners will be women who support the NPECDP-UKA implementation as translators and interpreters. They will contact the study participants during the intervention, motivating them to attend all workshops and the data collection during the whole duration of the study.

#### Data management

The data management team, based at the National Institute of Public Health, will elaborate capture masks in REDCap for e-tablets [36]. The data capture system will include automated skip patterns and data value range checks according to instrument structure. The data will be securely stored locally in tablets and then transferred to a centralized data management system with a data quality control protocol overseen by the lead data manager. Study staff will employ several strategies to promote data quality, including double data entry, and range checks for data values during study analyses and applying auditable algorithms for the systematization and automatic identification of possible errors in the values of the measured characteristics. Daily visual cross-validation of the data for complex errors, and regular on-site monitoring, the quality and completeness of the data will be reflective of the state of the trial.

#### Confidentiality

To protect participants’ confidentiality, participant data will be labeled using a unique participant identification code that contains no personal identifiers. Access to the collected participants’ data will be restricted to the principal investigator and appropriately trained Institutional Review Board (IRB)-approved research study staff as required. All laboratory samples, completed forms, reports, and other records will be identified using an unlinked unique participant ID number to maintain participant confidentiality.

#### Plans for collection, laboratory evaluation, and storage of biological specimens for genetic or molecular analysis in this trial/future use

In order to know the anemia status of children, hemoglobin will be measured every 3 months. Trained personnel will obtain capillary blood samples for the diagnosis and timely treatment of anemia in children between 6 months and 5 years and pregnant women. For the detection of anemia, the Hemocue Hb 201™ analyzer will be used. This analyzer provides a measurement of total hemoglobin in whole blood, capillary, venous, or arterial, with the same quality as a hematology analyzer. This system is designed for the quantitative determination of hemoglobin at the point of care in primary care areas and is for in vitro diagnostic use only. No storage and future use of this biological material will be needed.

### Statistical methods {20a, 20b, 20c}

#### Statistical methods for primary and secondary outcomes

The effects of the NPECDP-UKA on ECD for primary and secondary outcomes will be assessed by the difference of proportions for binary outcomes and the difference of means for quantitative outcomes. In case there are unbalanced observed characteristics across groups, effects will be estimated with logistic multiple regression for binary outcomes and with multiple linear regression for quantitative outcomes. Covariate-adjusted means or proportions will be obtained after model estimation as predictive margins [37]. Standard errors will be adjusted for clustered data using the method of linearization [33]. Additionally, the difference in difference estimators with propensity score matching will be performed to approximate effects with a quasi-experimental approach [23]. In this analysis, the unexposed group consists of children of caregivers who declined to participate in the NPECDP-UKA but acceded to participate in the evaluation study.

#### Interim analyses

No interim analyses will be performed. Analyses with measurements before the final data point will be performed only for subsamples or comparisons for which measurements will be completed by then: for example, the quasi-experimental part of the study or the concurrent validation analysis of the EDI-II test results. Therefore, no interim analyses will be used for deciding on study termination.

#### Methods for additional analyses

An analysis of the mediating role of parenting practices between intervention exposure and ECD will be carried out using structural equation models. Parameters will be estimated through weighted least-squares with mean and variance adjustment and the theta parameterization [38].

#### Methods in analysis to handle protocol non-adherence and any statistical methods to handle missing data

Complete case analysis will be performed as well as multiple imputation analysis when appropriate [39]. In regard to adherence, analyses will be complemented with a dose-response analysis.

#### Plans to give access to the full protocol, participant-level data, and statistical code

Full protocol and used code will be shared upon proper and formal request for academic reasons. Datasets are not public so access should be requested formally.

### Oversight and monitoring {21a, 21b, 22, 23}

#### Composition of the coordinating center and trial steering committee

The execution of the trial will be performed by UKA and its research department will function as the coordinating center. The steering committee will be composed of the study investigators and the head of the research department of UKA. The data management team will include IT experts from both UKA and the sponsor institution. At the field, UKA experts will be in charge of electronic data generation through specialized hardware and the InfoKilo v2 information system. IT experts from the National Institute of Public Health of Mexico will monitor data quality and provide advice and recommendations based on auditable algorithms developed for quality control of the data collected.

#### Composition of the data monitoring committee, its role, and reporting structure

The data monitoring committee will be presided by one investigator from the National Institute of Public Health of Mexico who will coordinate with the data management team to review data generating processes and their quality.

#### Adverse event reporting and harms

No unintended adverse effects are expected from the intervention; however, any adverse events related to the execution of the study will be reported to the supervisor in charge of the corresponding area who in turn will immediately report to the IRB.

#### Frequency and plans for auditing trial conduct

The principal investigator will designate appropriately qualified personnel to periodically perform quality assurance checks at mutually convenient times during and after the study and based on auditable algorithms that were developed for quality control of the data collected. These monitoring visits provide the opportunity to evaluate the progress of the study and the adherence to the intervention and obtain information about potential problems. Scheduling monitoring visits will be a function of participant enrollment, site status, and other commitments. The monitor will assure that data are accurate and in agreement with any paper source documentation used, verify that subjects’ consent for study participation has been properly obtained and documented, confirm that research subjects entered into the study meet inclusion and exclusion criteria, and verify that study procedures are being conducted according to the protocol guidelines. If a problem is identified during the visit (i.e., poor communication with the data coordinating center, inadequate or insufficient staff to conduct the study, etc.), the monitor will assist the site in resolving the issues. Some issues may require input from the IRB or of the principal investigators.

#### Plans for communicating important protocol amendments to relevant parties {25}

Protocol amendments will be submitted to the research committee of the National Institute of Public Health Mexico and when necessary and appropriate to the research ethics committee. Authorized changes will be submitted to the Clinical Trials profile of the study.

#### Dissemination plans {31a}

Plans for dissemination include national and international congresses, academic events. and peer-reviewed publications of results at different stages of the project.

## Discussion

We have presented an evaluation design to estimate the effect of a nurturing care intervention on ECD. Most common designs in evaluation are not applicable to estimating effects on ECD given the nature of the outcome. Scales used to assess dimensions of ECD depend on the specific age of subjects; comparing scores across time is problematic since the way in which ECD is measured varies with age. On the other hand, multiple sources of bias should be considered when selecting a design. The main sources of bias are due to confounding factors such as cohort effects, community effects, self-selection, aging effects, and period effects. Community effects can be controlled by randomization. This type of design is known as a cluster randomized trial [40], where the unit that receives the intervention is an aggregate unit, typically subjects are nested into community clusters. Another advantage of the cluster randomized trial design is related to potential spillover effects. For example, in an educational intervention, subjects that receive the intervention may communicate what they learn to neighbors.

Special consideration should be given to the number of communities to be randomized so that intervention effects may be properly separated from community effects. In the extreme case of just two clusters, even with a random allocation, effects from the intervention are totally confounded with the specific characteristics of the two communities. One of the communities may have better outcomes simply because of its own characteristics and not necessarily because of the applied intervention. It has been suggested a total of between ten and fifteen communities per arm [40] to better separate community effects from intervention effects. In the present study, we specified a total of twenty communities per arm.

The stepped-wedge design has been proposed for tackling limitations of classical designs when a control group is not feasible given ethical or logistical considerations [20]. Our design corresponds to a specific stepped-wedge design where effects are assessed as in a parallel design. The main feature of this design is the sequential incorporation of study groups; the defining characteristic of study groups is the time of exposure to an intervention. The assignment of experimental units to study groups is randomized and evaluation can be performed at the same calendar period across study groups; this characteristic precludes effects from time of measurement to be confounded with intervention effects. An alternative version of the stepped-wedge design proposes comparing measurements of the same group before and after intervention; since this occurs in different calendar times, confounding due to period effects cannot be ruled out under this setting [20].

Another type of confounding relates to age; given the nature of ECD outcomes, it is key to compare intervened and not intervened subjects at the same ages. This guarantees that the very same items from ECD scales are used to assess intervention effects. On the other hand, changes in child development at early ages occur very rapidly. This characteristic of ECD complicates using classical estimators such as the difference in differences estimator mainly for three reasons: (1) at the individual level, it is difficult to interpret changes when assessment items vary with age; (2) time differences between measurements across study groups are required to be balanced to avoid confounding; and (3) the distribution of initial ages should also be balanced across study groups. Although these imbalances may be attenuated by using adjustment covariates in models, it would be preferable to avoid these sources of potential bias with a robust design.

Another important source of bias, especially in programs that are not possible to randomize individuals for ethical reasons, is self-selection. Our study has a quasi-experimental component where self-selection is tackled analytically through propensity score matching techniques and difference in difference estimators. The experimental component of our design avoids self-selection bias since all subjects are self-selected to receive the intervention. The key difference between groups is the moment at which intervention is implemented. Groups are incorporated in stages; the last group incorporated is measured before the intervention starts so it serves as a comparison group. Effects are assessed as in a parallel design. In other contexts, the stepped-wedge design has been identified as a quasi-experimental approach; however, it has been noted that a well-conducted stepped-wedge trial where period effects are controlled and participants experience only one condition can in principle be as rigorous as a standard control randomized trial [41].

The intervention proposed share components with other interventions that have proved beneficial effects. Interventions that provide micronutrients for pregnant women and undernourished children have shown improvements in infant nutrition [16, 42, 43]. Also, interventions that include parenting counseling about proper diet and complementary and responsive feeding have showed benefits in the nutritional status of young children [44, 45]. Parent counseling on stimulation has been successful in improving ECD, and this counseling could be made by peers [46], through home visits [47] or workshops and parent sessions [48–51].

Our proposed design has its own limitations that include vulnerability to exogenous shocks that may compromise effects estimation. Although random allocation of communities to the order of intervention implementation balances (in expected value) observed and unobserved characteristics across study groups, benefits of realized interventions could be lower compared to what would be obtained in a situation without an external shock.

Our study design is recommended when the way in which outcomes are measured depends on age, self-selection is present, and assignment is performed at an aggregate level. Although key sources or biases are avoided (e.g., randomization within blocks guarantees that community characteristics that were used to define blocks are balanced between study groups), implementing our design may be challenging given its required sample size and the coordination efforts necessary.

According to our knowledge, this is the first experimental study on ECD in Mexico and the Latin American region which will evaluate a social program designed by a Mexican non-governmental organization, aimed at impacting neurological development through the improvement of child rearing practices. Likewise, this study will allow to generate robust and rigorous information on the causal mechanisms that determine the achievements in neurodevelopment in contexts of high social vulnerability, and this will be useful for the design and implementation of effective ECD interventions.

## Trial status

Recruitment started in July 2019 and was scheduled to end in June 2022. During the first year of the study, once potential participants had been identified, researchers conducted two recruitment phases: the first one from July 15 to December 19, 2019, and the second one from January 21 to February 10, 2020. Due to the COVID-19 public health emergency, recruitment was suspended during early March 2020. Baseline measurements were obtained for a total of 1176 children (764 whose caregivers decided to enroll and 412 whose caregivers decided not to enroll in the NPECDP-UKA).

## Data Availability

The dataset will be available upon reasonable request once the results have been published. The request will be evaluated by the PI to ensure that it meets reasonable standards of scientific integrity and has the potential to make a reasonable scientific contribution.

## Abbreviations

INSP: National Institute of Public Health of Mexico (Spanish)
ECD: Early childhood development
NGOs: Non-governmental organizations
UKA: Un Kilo de Ayuda A. C.
NPECDP-UKA: Neurological and Psycho-affective Early Childhood Development Program
CDE-II: Child Development Evaluation Test 2nd Edition
EDI-II: Evaluación del Desarrollo Infantil
WHO: World Health Organization
MSCA: McCarthy Scales of Children’s Abilities
GCI: General Cognitive Index
BSID-III: Scales of Infant and Toddler Development, Third Edition
